# Large Chorioangioma in Triplets: An Uncommon Occurrence

**DOI:** 10.7759/cureus.40790

**Published:** 2023-06-22

**Authors:** Avin Kounsal, Divya Saini, Vivek Podder, Chetan Mehta, Pokhraj P Suthar

**Affiliations:** 1 Department of Diagnostic Radiology and Nuclear Medicine, Rush University Medical Center, Chicago, USA; 2 Department of Public Health, Johns Hopkins Bloomberg School of Public Health, Baltimore, USA; 3 Department of Medicine, Bangladesh Institute of Research and Rehabilitation in Diabetes, Endocrine and Metabolic Disorder, Dhaka, BGD; 4 Department of Radio-Diagnosis, Sir Sayajirao General (SSG) Hospital and Medical College Baroda, Vadodara, IND

**Keywords:** placenta, obs, sonography, triplets, chorioangioma

## Abstract

A 25-year-old primigravida presented at 26 weeks of gestation by dates, the first time for the routine antenatal checkup. No histories were suggestive of pregnancy-induced hypertension (PIH) and edema. On physical examination, pallor was present with microcytic hypochromic anemia. Raised beta-human chorionic gonadotropin (HCG) and alpha-fetoprotein (AFP) levels were present. Ultrasonography revealed triples with two thin echogenic intertwining membranes. Anomaly scan did not reveal any abnormality in fetuses. The placenta showed a large oval hypoechoic mass arising from its edge and bulge into the amniotic fluid. A central feeding vessel with a branching pattern and pulsatile color flow like that of the umbilical artery is noted on the color Doppler. She was spontaneously preterm delivered vaginally at 28 weeks of gestation. All three fetuses were stillborn. Histopathological diagnosis of angiomatous chorioangioma was confirmed. This case classically represents a grave complication of the large chorioangioma.

## Introduction

A placental chorioangioma is a benign vascular tumor of placental origin. It is the most common tumor of the placenta, with an estimated incidence of 0.6% of all pregnancies [1]. The risk is increased after multiple pregnancies and in female babies [2]. Small chorioangiomas are usually asymptomatic, while large chorioangiomas are associated with high perinatal morbidity and mortality and a poor prognosis [2]. Ultrasound (USG) with color duplex is a non-invasive imaging modality that helps diagnose placental chorioangioma. In this case report, we present a 25-year-old primigravida with an incidental diagnosis of chorioangioma on ultrasound.

## Case presentation

A 25-year-old primigravida presented at 26 weeks of gestation for the first time for a routine antenatal checkup. No histories were suggestive of pregnancy-induced hypertension or edema. Her vitals were within normal range (blood pressure of 110/80 mm Hg, respiratory rate of 16/min, and pulse rate of 84/min). Except for pallor, the rest of the physical examination was unremarkable. The patient’s height was 156 cm, and her weight was 58 kg. Her hemoglobin (Hb) was 8.0 g/dL with a peripheral smear suggestive of microcytic hypochromic anemia. Her serum β-human chorionic gonadotropin (HCG) and alpha-fetoprotein (AFP) levels were elevated (393,514 IU and 620 ng/mL, respectively). A urine examination for albumin and glucose was negative. HIV and venereal disease research laboratory (VDRL) tests for syphilis were negative. The patient was then referred to the Department of Radiology for a routine antenatal ultrasound.

All ultrasound examinations were performed using a color Doppler duplex ultrasound system equipped with a 3.75-15 MHz transducer. Ultrasonography revealed triples (three fetuses) with USG parametric measurements, as illustrated in Table 1. Lie of the fetuses were variable.

**Table 1:** Ultrasonographic parametric measurements of the fetuses. BPD: biparietal diameter, HC: head circumference, FL: femur length, AC: abdominal circumference, GA–AUA: gestational age–actual ultrasound age.

	Fetus A	Fetus B	Fetus C
BPD	26 weeks, 0 days (6.44 cm)	25 weeks, 5 days (6.35 cm)	25 weeks, 2 days (6.25 cm)
HC	25 weeks, 6 days (23.76 cm)	26 weeks, 0 days (23.94 cm)	25 weeks, 4 days (23.57 cm)
FL	25 weeks, 6 days (4.72 cm)	26 weeks, 2 days (4.83 cm)	26 weeks, 0 days (4.77 cm)
AC	25 weeks, 6 days (21.34 cm)	26 weeks, 1 day (21.66 cm)	25 weeks, 3 days (20.94 cm)
GA–AUA	25 weeks, 6 days	26 weeks, 0 days	25 weeks, 4 days

The fetal fundic bubble, kidneys, bladder, four-chamber view of the heart, spine, and cranial sonography of all fetuses were normal. There was no pleural effusion or ascites in any of the fetuses. Three vessels of the umbilical cord were present. Polyhydramnios was present in all three fetuses. Two thin echogenic intertwining membranes were present between the fetuses. There were three placentas, and a well-defined oval hypoechoic mass measuring 10.3 × 5.8 cm was seen at the edge of the placenta, causing a lobulated bulge into the amniotic fluid. On application of the color Doppler imaging, a central feeding vessel with a branching pattern was seen, and on the pulsed Doppler, it showed a pulsatile flow similar to that of the umbilical artery.

**Figure 1:**
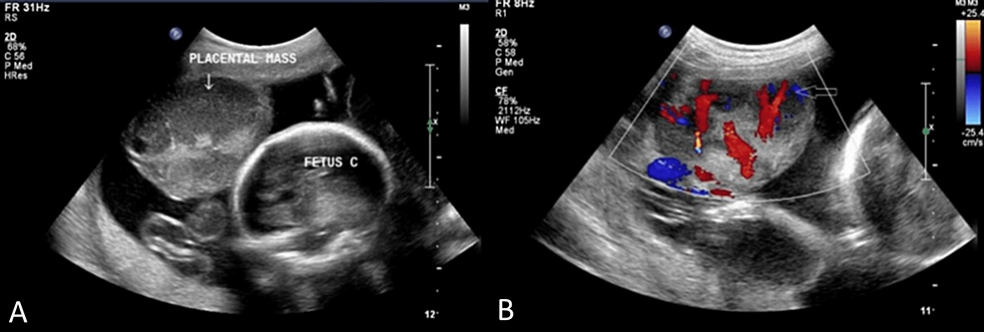
(A) An ultrasonographic image with a 5 MHz curvilinear probe shows a well-defined hypoechoic mass lesion (solid white arrow) arising from the edge of the placenta and protruding into the amniotic cavity. Three vessels in the umbilical cord are seen. (B) A color Doppler image with a 5 MHz curvilinear probe shows vascularity within the hypoechoic placental mass lesion (open white arrow).

**Figure 2:**
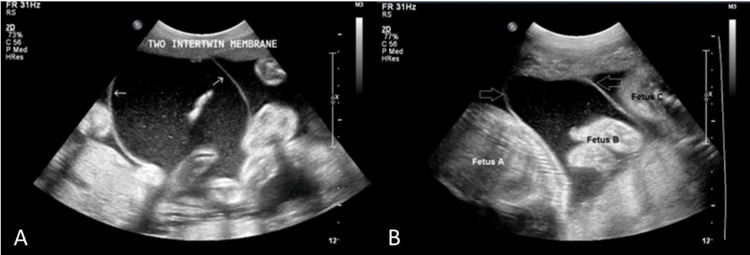
An ultrasonographic image shows two echogenic, intertwined membranes (white arrows in (A) and open white arrows in (B)) among three fetuses.

The diagnosis of chorioangioma in the triplets was postulated based on the ultrasonographic and color Doppler findings (Figures 1, 2). The patient was advised to adhere to absolute bed rest and receive weekly checkups. The follow-up course was unrevealing, without any complaints. The patient was spontaneously preterm and delivered vaginally at 28 weeks of gestation. All three fetuses were stillborn. The placenta contained a lobular gray and tan-colored mass measuring 10 × 6 cm. Histopathology revealed numerous small areas of endothelial tissue, capillaries, and blood vessels surrounded by placental stroma. A histopathological diagnosis of angiomatous chorioangioma was confirmed.

## Discussion

A placental chorioangioma is a benign vascular tumor of placental origin and is the most common tumor of the placenta. It was first described by Clarke in 1798 [3]. The estimated incidence is 0.6% of all pregnancies [1]. A chorioangioma consists of a benign angioma arising from chorionic tissue and increases in risk after multiple pregnancies and in female babies.

There are three histological patterns of chorioangiomas: angiomatous, cellular, and degenerative [4]. Angiomatous is common with good vascularization, and cellular is common with poor vascularization. Chorioangiomas vary significantly in size, but there is no malignant potential associated with them [5]. Small lesions are common, and lesions larger than 4 cm are rare [6]. Degenerative changes, such as necrosis, hyalinization, myxomatous changes, and calcification, are seen in large chorioangiomas.

In most cases, chorioangiomas are asymptomatic and are diagnosed as incidental. However, poor outcomes for both the fetus and the mother are seen with multiple and large chorioangiomas. Complications for the fetus include developing high cardiac output failure and hydrops fetalis due to arterio-venous shunting [7], and fetal thrombocytopenia. Complications for the mother include preterm labor, polyhydramnios, pre-eclampsia, and intrauterine growth retardation. Further, raised maternal AFP, single umbilical artery, and fetal anemia are associated with chorioangiomas.

Chorioangiomas occur near the insertion site of the umbilical cord and protrude into the amniotic cavity. On grayscale ultrasonography, chorioangiomas are rounded hypoechoic masses seen near the chorionic plate or at the insertion site of the umbilical cord. Anechoic cystic areas may be seen within. Heterogeneous areas within it represent degenerative changes and internal hemorrhage. A pedunculated appearance is rarely seen. Pulsatile color flow within the anechoic cystic area on color Doppler images represents enlarged vascular channels. Spontaneous infarction is sometimes noted in large chorioangiomas as decreased tumor volume, decreased echogenicity, and decreased blood flow on color Doppler images [8]. On T1-weighted MRI scans, chorioangiomas are isointense to the placenta if they are uncomplicated. A hyperintense signal represents a hemorrhage within.

Chorioangiomas should be differentiated from partial hydatidiform moles, degenerated uterine fibroids, placental teratomas, and blood clots on USG and color Doppler images, as illustrated in Table 2.

**Table 2:** Differential diagnosis of chorioangiomas on USG and color Doppler. USG: ultrasound.

	USG and color Doppler
Partial hydatidiform mole	Absence of vascular channels similar to fetal vessels
Degenerated uterine fibroid	Seen toward the maternal surface and the absence of vascular channels similar to fetal vessels
Placental teratoma	Absence of vascular channels similar to fetal vessels
Blood clots	The echo pattern of the clots differs with time, while the chorioangioma remains the same

Small chorioangiomas are often monitored with ultrasound every six to eight weeks, whereas large chorioangiomas require weekly USG examinations. Chorioangiomas may regress spontaneously during pregnancy. However, complicated chorioangiomas appearing before fetal viability require intervention. Various techniques, such as serial fetal transfusions and fetoscopy laser coagulation of vessels supplying the tumor, may be effective [9,10]. In our case, therapeutic options were limited as this case occurred in a rural part of India. Therapeutic amniocentesis and maternal indomethacin therapy are useful for polyhydramnios. Steroid administration before 34 weeks is recommended to accelerate fetal lung maturity. Large chorioangiomas are associated with high perinatal morbidity and mortality and poor prognoses.

## Conclusions

A placental chorioangioma is the most common benign vascular tumor of placental origin. The risk is increased after multiple pregnancies and in female babies. Antenatal diagnosis is achieved by ultrasound, and Doppler is the investigation method of choice for diagnosing chorioangiomas and ruling out other causes of placental masses. Large chorioangiomas are associated with high perinatal morbidity and mortality as well as poor prognoses, which warrants institutional and timely delivery. This case with classical ultrasound findings will raise awareness in the reader about rare entity. 
